# Plants do not count… or do they? New perspectives on the universality of senescence

**DOI:** 10.1111/1365-2745.12089

**Published:** 2013-04-24

**Authors:** Roberto Salguero-Gómez, Richard P Shefferson, Michael J Hutchings

**Affiliations:** 1 Evolutionary Biodemography Laboratory, Max Planck Institute for Demographic ResearchKonrad-Zuße straße 1, 18057, Rostock, Germany; 2 Centre for Biodiversity and Conservation Science, University of QueenslandGoddard Building #8, St Lucia, Qld, 4072, Australia; 3 Odum School of Ecology, University of Georgia140 East Green Street, Athens, GA, 30601, USA; 4 School of Life Sciences, University of SussexFalmer, Brighton, Sussex, BN1 9QG, UK

**Keywords:** ageing, antagonistic pleiotropy, comparative plant demography, disposable soma, dormancy, longevity, mutation accumulation, oxidative stress, plant development and life-history traits, senescence

## Abstract

**1.** Senescence, the physiological decline that results in decreasing survival and/or reproduction with age, remains one of the most perplexing topics in biology. Most theories explaining the evolution of senescence (i.e. antagonistic pleiotropy, accumulation of mutations, disposable soma) were developed decades ago. Even though these theories have implicitly focused on unitary animals, they have also been used as the foundation from which the universality of senescence across the tree of life is assumed.

**2.** Surprisingly, little is known about the general patterns, causes and consequences of whole-individual senescence in the plant kingdom. There are important differences between plants and most animals, including modular architecture, the absence of early determination of cell lines between the soma and gametes, and cellular division that does not always shorten telomere length. These characteristics violate the basic assumptions of the classical theories of senescence and therefore call the generality of senescence theories into question.

**3.** This Special Feature contributes to the field of whole-individual plant senescence with five research articles addressing topics ranging from physiology to demographic modelling and comparative analyses. These articles critically examine the basic assumptions of senescence theories such as age-specific gene action, the evolution of senescence regardless of the organism's architecture and environmental filtering, and the role of abiotic agents on mortality trajectories.

**4.** *Synthesis*. Understanding the conditions under which senescence has evolved is of general importance across biology, ecology, evolution, conservation biology, medicine, gerontology, law and social sciences. The question ‘*why is senescence universal or why is it not?’* naturally calls for an evolutionary perspective. Senescence is a puzzling phenomenon, and new insights will be gained by uniting methods, theories and observations from formal demography, animal demography and plant population ecology. Plants are more amenable than animals to experiments investigating senescence, and there is a wealth of published plant demographic data that enable interpretation of experimental results in the context of their full life cycles. It is time to make plants count in the field of senescence.

Age is an issue of mind over matter. If you don't mind, it doesn't matter

Mark Twain (1835–1910).

## Introduction

Complex organisms such as giant redwood trees, orchids and humans have come to be as a result of natural selection. Despite the optimizing impact of natural selection, even these complex species do not seem capable of indefinite self-maintenance, and so the Darwinian demon (Law 1979) has not evolved (Mitteldorf 2006; Libertini 2008). If an organism is not killed by external factors (e.g. fire, predation, etc.), life-history theory predicts decline in its physiological functions at advanced ages, which ultimately results in structural collapse and death. This biological process – *senescence* – is widely recognized as one of the most puzzling phenomena in evolutionary biology (Sherrat & Wilkinson 2009; Baudisch & Vaupel 2012).

In principle, a phenomenon such as senescence, which has a negative effect on the fitness of an organism, should be eliminated by natural selection. But if this is the case, why is senescence so widely observed in nature [but mostly in animals; see *Taxonomic Bias* below (Holliday 2006; Monagham *et al*. 2008; )]? Theories to explain this phenomenon include (i) the accumulation over evolutionary time of negative mutations at much higher rates late in life than early in life (*mutation accumulation*; Medawar 1952), (ii) the existence of trade-offs in gene expression between beneficial effects early in life vs. detrimental effects late in life (*antagonistic pleiotropy;* Williams 1957) and (iii) the confluence of trade-offs between survival and reproduction on the one hand, and limiting resources on the other, which inevitably leads to physiological deterioration late in life (*disposable soma*; Kirkwood 1977). All of these theories have as a common feature the prediction that senescence evolves in eukaryotic, iteroparous species due to the decreasing force of selection in older individuals. In other words, the remaining reproductive value of old individuals cannot match that of young individuals, and so even strong selection against the traits of older individuals has little evolutionary impact.

Most classical theories of senescence were developed with an implicit general animal (e.g. mammals in Medawar 1952; Promislow & Harvey 1990; birds in Ricklefs 2000) or explicit human bias in mind (Medawar 1952; Maier *et al*. 2010). In spite of taxonomic biases, understandably driven by an anthropocentric interest in delaying death (Bartke *et al*. 2001; Maier *et al*. 2010) and improving life quality at advanced ages (Crews 2003), the claim has been made that senescence is universal. Hamilton (1996, p. 20) stated that senescence should occur even ‘in the farthest reaches of almost any bizarre universe’. His assertion is of broad interest to evolutionary biologists in general and plant ecologists in particular because (i) it suggests the existence of a universal rule of ecology and evolution that is yet to be tested, and (ii) it obviously suggests that plants are not immune from senescence.

The universality of senescence has been questioned recently (Baudisch 2005; Steinsaltz, Evans & Wachter 2005). Mathematically, Vaupel *et al*. (2004) have argued that senescence might be avoided by organisms with indeterminate growth (e.g. some reptiles, most fish, many molluscs, fungi and plants), where attributes such as size are typically better predictors of population dynamics than age (Caswell 2001). This mathematical treatment suggests in particular that senescence may be avoided if small decreases in current reproduction can yield large increases to life span and future reproduction (Vaupel *et al*. 2004). The somewhat limited exploration of senescence that has been conducted in the plant kingdom shows a variety of results, ranging from support for the existence of senescence (Nooden 1988; Watkinson 1992; Roach 1993; Pedersen 1999; Thomas 2003), to no significant changes in fitness components through chronological age (*negligible senescence*; Piñero, Martínez-Ramos & Sarukhan 1984; Finch 1990; Bond 2000; Lanner & Connor 2001), to increasing survival and fecundity with age (*negative senescence sensu* Vaupel *et al*. 2004; see García, Dahlgren & Ehrlén 2011).

This Special Feature, entitled ‘New Perspectives in Whole-Plant Senescence’, offers a broad overview on the current state of understanding of whole-plant senescence. Our goals are (i) to introduce the currently rich evolutionary and ecological research agenda of the exploration of senescence in the plant kingdom, (ii) to briefly document how the exploration of senescence can benefit plant ecological research and (iii) to suggest future directions in its study. Ultimately, we hope that the collection of papers in this Special Feature will encourage the development of new, more comprehensive theories of senescence, their explicit testing using experimental manipulations in plants and the establishment of links with animal demographers to address the evolution of senescence using a truly comparative framework.

## Whole-plant senescence: terminology, taxonomic bias and ecological implications

As in other disciplines in biology, the field of senescence has not escaped the application of overly complicated, often confusing terminology. It is not our wish or role here to discuss the etymology of the terms senescence and ageing, or their correct usage. Instead, we and the authors in this Special Feature use the term senescence to refer to a ‘decline in age-specific fitness components (i.e. survivorship and reproduction) due to internal physiological deterioration’ (Rose 1991). The term ageing is used to refer to the mere accumulation of years by the individual, conditional on its survival. Thus, in what follows, (chronological) ageing, unlike senescence, does not inform on change in the fitness components of an individual.

In plant-based literature, it is common to find the term senescence applied to the phases prior to and during the abscission of organs [e.g. leaves (Thomas & Stoddart 1980; Jing & Nam 2012), fruits (Nooden 1988), roots (Fisher, Eissenstat & Lynch 2002; Watteau *et al*. 2002) or other plant parts (Kozlowski 1973)]. Unlike the literature on whole-plant senescence, the research on organ senescence is vast. A search (31 January 2013) in ISI Web of Knowledge using the keywords ‘(senescence OR aging OR ageing) AND plant’ produced 31 315 hits. A subsample of this literature (*n* = 500 papers) revealed that < 4% addressed senescence at the whole-plant level. Senescence of leaves and/or the genetic machinery of plant senescence have been recently treated in other Special Features (Beck & Scheibe 2003; Balazadeh *et al*. 2008; Jing & Nam 2012) and reviews (Thomas 2012). Here, we focus on the whole organism, which is at the biological level of organization that is most relevant to the ecological and evolutionary consequences of senescence, including relationships to plant ecophysiological and demographic traits.

The current landscape of research into whole-plant senescence appears rather deserted. This is surprising, because declines in whole-plant fitness with age have obvious consequences for all branches of plant biology. Senescence can contribute to a decline in productivity of many economically important crop plants (Pate 1988; Thomas 1992). Less obvious consequences of senescence also touch on our understanding of plant ecology, including plant ecophysiology, plant population ecology, community ecology and conservation biology. The following is a short list of less straightforward yet important connections between plant senescence and the aforementioned fields. It is not our intention to offer a thorough list of all connections, but rather to indicate the relevance of understanding senescence to some of the most fundamental questions in ecology (Sutherland *et al*. 2013), and to elucidate how the analysis of age-based trajectories of survivorship and reproduction in plants can aid our research.

- Allometric scaling, whereby basic individual characteristics such as biomass are used as proxies to understand the vast variation in life spans among species, has been the focus of some very active research in recent decades. This body of literature predicts mortality rates to scale as the -¼ power of individual size across the tree of life (West, Brown & Enquist 1997; Enquist *et al*. 1999). Nonetheless, significant uncertainty surrounds the *R*^2^ values obtained in this type of correlative analyses, and this may be due in part to the way in which age-based trends are confounded by size in plants. Incorporating both age and size into analyses under this framework may be useful, and once carried out, examination of the *shape* of the mortality curve (*sensu* Baudisch 2011), and the speed (*pace*) at which life goes by, will add new insights and likely tighten our understanding of these relationships.
- Dendroecologists examine ring chronology of extremely long-lived specimens of species such as bristlecone pine (*Pinus longaeva*; Fritts & Swetnam 1989) and giant sequoia (*Sequoiadendron giganteum*; Swetnam 1993) to look back in time. Variations in the width between annual rings are typically used to retrospectively infer differences in precipitation, temperature or fire events over time. These studies ignore the fact that trees may lose vigour with age (Mencuccini *et al*. 2005, 2007) and that therefore the carbon accumulated between rings may also be affected by senescence. We know of no dendroecological study that has corrected these retrospective insights for rates of senescence. Including senescence in dendroecological research could change the interpretation of past events.
- Ecophysiologists have recently become interested in the way in which the internal organization of a plant is influenced by abiotic conditions. Vascular plants are internally organized into a collection of repeated physiological units (*sensu* Watson & Casper 1984). These units may share more or less water, minerals and carbohydrates depending on characteristics such as neighbouring distance, amount of heartwood or xylem lumen (Holbrook & Zwieniecki 2005). Schenk *et al*. (2008) discovered that the degree of hydraulic sectoriality, which determines the extent to which physiological units are likely to share resources, increases with aridity. Similar patterns and processes of resource sharing have been reported in modules of clonal plants (Price, Hutchings & Marshall 1996; de Kroon & van Groenendael 1997; Hutchings 1999; Alpert, Holzapfel & Slominski 2003). Understanding how the physiology of plants underlies their demographic rates of senescence will undoubtedly help to explain why some of the longest-lived plant species are found in extremely arid areas (Bowers, Webb & Rondeau 1995; Lanner & Connor 2001; Peñuelas & Munné-Bosch 2010).
- Plant population ecologists often make the assumption in building demographic models that ‘bigger is better’. In other words, larger individuals are *putative* mothers of more recruits the next year. However, large plants may not necessarily produce more seeds, nor may they necessarily have greater survival than smaller individuals (Bierzychudek 1982). Nevertheless, assumptions of this type are common in plant population ecology, probably due to field sampling convenience (e.g. Valverde & Silvertown 1998; Barot & Gignoux 1999) and the absence of extensive genetic markers for species apart from *Arabidopsis*. If larger individuals of a plant population undergo reproductive senescence, whereby their seed and/or flower production per capita is lower than that of younger, smaller individuals (Enright & Watson 1992), extrapolations to classical perturbation analyses may be flawed.
- Community ecologists have long highlighted the tight, complex connections, or networks, that provide the necessary flows of energy for sustainability among co-occurring species. The balance of a whole network can be strongly affected by the perturbation of a single, keystone species (Saavedra *et al*. 2001; Stouffer *et al*. 2012). For instance, shifts in peak flowering time associated with raising temperatures as a consequence of global warming have been found to create phenological mismatches within mutualistic networks (Hegland *et al*. 2009). For two species showing reproductive senescence in the form of lower seed production during the life span of the individuals (*Plantago lanceolata* in Lacey *et al*. 2003; *Beta vulgaris spp. maritima* in van Dijk 2009), delays in peak flowering time also occurred with age every flowering season. The extent to which the age structure of the population interacts with shifts in overall peak flowering time due to climate change is yet to be explored. It is entirely possible that senescence may contribute to the disruption of mutualistic networks in unforeseen ways.
- Senescence can also affect community diversity and composition. Bowers & Turner (2001) showed that intrinsic age patterns of mortality in the desert tree *Cercidium microphyllum* can interact with natural droughts to reverse competitive–facilitative relationships involving the saguaro cactus (*Carnegiea gigantea*). Likewise, the recent global decline in number of old trees has become a major concern, since they provide unique, important services for ecosystem integrity and biodiversity (Lindenmayer, Laurance & Jerry 2012).
- From a conservation biology perspective, extremely long-lived organisms, such as Bristlecone pine, sequoias or the saguaro cactus, are charismatic and aesthetically pleasing to humans. Long-lived redwoods have become flagship species for conservation in California, drawing considerable attention to the need for preservation of suitable habitats for their survival (Ferguson 1968). A better understanding of their biology, including why they seem to ‘escape’ senescence, can only enhance the public's appreciation of them.

## Contributions of the special feature

The papers in this Special Feature are intentionally biased towards the exploration of senescence as a universal phenomenon and written with the intention of promoting the understanding of its underlying causes. They use innovative methodological approaches to analyse remarkable data sets in the plant kingdom and yield valuable and novel insights into the biology of ageing.

Several recent publications have highlighted the need for a better understanding of the causes of senescence (García, Dahlgren & Ehrlén 2011; Baudisch & Vaupel 2012; Roach 2012). The contribution by Morales *et al*. (2013) takes the recent report of negative senescence in the dioecious, herbaceous perennial *Borderea pyrenaica* (García, Dahlgren & Ehrlén 2011) one step further. The authors explore photo-oxidative stress markers (Foyer, Lelandais & Kunert 1994) in individuals of this species for a range of ages (up to *ca*. 250 years old), sizes and sexes. Morales *et al*. found that chlorophyll levels, *F*_v_/*F*_m_ ratio and lipid peroxidation, all of which are proxies for oxidative damage, remained constant regardless of sex and age, suggesting the absence of age-associated oxidative stress at the organismal level. Furthermore, old female plants showed higher resilience to a natural drought than younger females, males or juveniles. These results are puzzling since female plants are expected to carry higher reproductive costs than either males or nonreproductive individuals (Obeso 2002).

Plants have remarkably complex life cycles. Some species set seed that may remain viable below-ground for over hundreds of years (Shen-Miller *et al*. 1995); some species can propagate new modules (ramets) seemingly indefinitely *via* clonality (Caswell 1985; Orive 1995; de Kroon & van Groenendael 1997). Perhaps the most remarkable aspect of the plant life cycle is the ability of some species to die back completely following one or more seasons of above-ground growth to spend prolonged periods of time – potentially several years in length – below-ground (vegetative dormancy; Shefferson *et al*. 2003; Shefferson 2009). All of the aforementioned processes might interfere with the way in which individual plants ‘count’ years. Tuomi *et al*. (2013) explore the interaction between vegetative dormancy and senescence in two extraordinarily long-term data sets for the perennial herbs *Astragalus scaphoides* and *Silene spaldingii*. The authors test the interactive effects between age and stage (stage in this case represented by dormant versus nondormant) in the life cycle on reproductive value – the integrative measure of fitness as a function of the age of the individual – in these species. Of the various models tested, one in which time progresses continuously from seed emergence to death was outperformed by other models that assumed that dormancy slowed or even reversed senescence. This study adds to a number of cases undermining the universality of senescence, by finding positive senescence in *A. scaphoides*, but negative senescence in *S. spaldingii*.

Another complex aspect of plant life histories is the ability of many species to fluctuate considerably in size, both positively and negatively, between years. Some herbs may shrink by up to 80% in just 1 year (Salguero-Gómez & Casper 2010; Salguero-Gómez *et al*. 2012), or grow substantially faster than the average individual in the population (Jansen *et al*. 2012). This phenomenon makes it impossible to estimate age directly from size or development because individual shrinkage reorganizes the structure of the population independently of age (Salguero-Gómez & Casper 2010) and developmental stage (Salguero-Gómez & Casper 2011). Shefferson and Roach test for size-based, age-indeterminate senescence in the herb *Plantago lanceolata* using over 10 years of data from plants allocated to four separate cohorts established in consecutive years. They use perturbation analyses (Caswell 2001) and invasion analysis (Metz, Nisbet & Geritz 1992) on age × size population matrices to show that the force of selection indeed decreases with age as predicted by Hamilton (1966). They then employ reverse age analysis (Martin & Festa-Bianchet 2011) to test whether the last few years of life are still dominated by declining physiological condition in a species in which actuarial senescence has never been documented. Their analyses show that individuals typically exhibit a decline in size over a period of three years prior to death, accompanied by lower inflorescence production. In their study, declines in physiological vigour are recorded prior to death. These declines in vigour are best explained by size rather than age, suggesting an important role for the environment in determining senescence.

The interactive effects of age and size are explored in depth from a mathematical perspective by Caswell & Salguero-Gómez (2013). The authors introduce the latest developments for analysis and decomposition of evolutionary pressures based on size/developmental stage, age and their interaction, for a set of 36 plant species ranging in growth form from bryophytes to trees. The methods introduced – an extension of the formulae for selection gradients of mortality described in Caswell (2012) – include selection gradients for fecundity as a function of age, size/developmental stage and their interaction. This study reports selection pressures on senescence that are fundamentally different from those expected by theories involving only classifications by age. The authors find life periods characterized by a senescent behaviour, but also other periods characterized by negative senescence in most sizes/developmental stages and species examined.

The comparative phylogenetic method is also used in this Special Feature to explore senescence in the plant kingdom. Baudisch *et al*. (2013) draw from published demographic information in the form of projection matrices, archived in the ComPADRe III data base (Salguero-Gómez 2013) to explore the validity of the statement of the universality of senescence put forward by Hamilton (1966, 1996). Baudisch and her collaborators use data from *ca*. 300 angiosperm species to survey patterns of senescence along two different dimensions of ageing – the length of life spans and the age pattern of mortality (pace and shape *sensu* Baudisch 2011) – and their correlations with habitat, growth form and phylogenetic ancestry. The study revealed that the majority of species show negligible or even negative senescence and that this result holds both for short- and long-lived species. Growth form significantly correlated with senescence rates (e.g. trees were more likely to display senescence than were other growth forms), whereas the shape of the mortality curves was strongly determined by phylogeny.

## Future directions in whole-individual senescence: making plants count

The implications of patterns of senescence at the whole-plant level radiate from evolutionary biology into most – if not all – sub-disciplines of plant ecology. Given the diversity of potential mechanisms that may cause senescence (Medawar 1952; Williams 1957; Hamilton 1966, 1996; Vaupel *et al*. 2004; Mitteldorf 2006; Libertini 2008; Baudisch & Vaupel 2012) and the conflicting evidence of its generality in the plant kingdom (Roach 1993; Thomas 2012), we have outlined 10 of the most fundamental questions in the study of senescence for which plants are particularly well-suited study organisms:

### 1. How May Intrinsic Causes of Mortality (i.e. Senescence) Be Distinguished from Extrinsic Causes of Mortality?

Senescence is generally assumed to be controlled by intrinsic factors, most notably the accumulation of alleles with negative effects in late life (Hamilton 1966) or trade-offs between maintenance and reproduction that make reproduction more important as maintenance becomes more costly in old age (Kirkwood 1977). Organisms in the wild are assumed not to exhibit senescence because most of them are killed by extrinsic processes before becoming old enough to do so (Promislow & Harvey 1990; Ricklefs 2000). Silvertown, Franco & Pérez-Ishiwara (2001) suggested that this mortality pattern affects herbaceous perennial species particularly. Indeed, it is difficult to distinguish death caused by intrinsic *vs*. extrinsic causes because senescence itself makes old individuals more susceptible to external forces of mortality (Mueller-Dombois 1987), even under controlled conditions. Whereas some authors have stated that making this distinction is virtually impossible (Kirkwood & Austad 2000), labour-intensive approaches such as the multi-cohort study on *Plantago lanceolata* (Roach 2009; Shefferson & Roach 2012, 2013) have been successful at disentangling not only intrinsic and extrinsic factors of mortality, but also the role of genetics. New statistical approaches using Bayesian and multivariate techniques may also facilitate research on this topic (Colchero, Jones & Rebke 2012; Holzwarth *et al*. 2013).

### 2. Does Physiological Rejuvenation Lead to Demographic Rejuvenation?

The universality of senescence rests on the assumption that the wear-and-tear of life is cumulative and inescapable over an organism's life span because time flows only in one direction (Charnov 1993; but see Tuomi *et al*. 2013). Yet, plants show extreme plasticity, being able to retrogress to juvenile stages under specific conditions. Chen *et al*. (2012) recently showed that the genetic and physiological activity of grafted stems of *Sequoia sempervirens* is the same as that in juveniles and very distinct from that of ungrafted adults. Plants have been historically considered as populations of modules (Harper 1977) in a continuous state of renewal and replacement, allowing continuous whole-plant rejuvenation. The relationship between leaf senescence, module senescence and whole-plant senescence remains largely unexplored (Roach 1993), and yet full of potential. For instance, many species (e.g. the orchid *Spiranthes spiralis*; Wells 1981) completely renew their photosynthetic and below-ground storage tissues annually. These species are potentially in a state of ‘perpetual somatic youth’ (*sensu* Harper 1977).

### 3. How Plastic Are Longevity and Senescence Rates in Plants, and How Will They Be Affected by Climate Change?

The advent of modern health care and other advances have enabled humans to delay senescence and prolong life span (Vaupel 2010). Global change, including increased temperatures and nitrogen deposition, and less predictable precipitation, is widely accepted as the largest set of threats to the earth's biota and is certainly influenced by human demography. The question remains as to how these changes will shape the mortality and fecundity trajectories of plants.

### 4. Does Dietary Restriction Affect Senescence in Plants in the Same Way as It Affects Animals?

A comparative study (Blagosklonny & Hall 2009) has suggested that the link between growth and senescence is nutritional in nature. Dietary restriction, involving decreased nutritional intake, has been reported to improve health and result in longer life span in animals (Weindruch *et al*. 1986; but see Phelan & Rose 2005; Mattison *et al*. 2012). Plant growth is limited by cell division, which depends on water turgor and mineral availability. Is it a coincidence that many of the longest-lived plant species are found in deserts, where water is most limiting and growth rates are among the lowest observed within the plant kingdom?

### 5. How Does Gender Affect Senescence in Plants?

Differences between sexes in performance at specific ages have been a focus of animal ecology for decades (Owens 2002 and references within), allowing for the exploration of connections between trade-offs, resource allocation and senescence. The work on the herb *Borderea pyrenaica* (García, Dahlgren & Ehrlén 2011; Morales *et al*. 2013) highlights, for the first time to our knowledge, differences between sexes in the rates of age-based fecundity and mortality in plants. An ideal candidate for further exploration of this question is *Arisaema triphyllum*, where changes in sex can take place through the life of individual plants (Lovett Doust, Lovett Doust & Turi 1986).

### 6. What Growth Forms, Environmental Pressures and Phylogenetic Background Predispose a Plant Species to Evolve or Escape Senescence?

The comparative work initiated by Silvertown, Franco & Pérez-Ishiwara (2001), and here expanded by Baudisch *et al*. (2013) has only explored the tip of the iceberg. We predict that the wealth of demographic data available in the plant kingdom (Salguero-Gómez 2013), and the relative ease with which plants can be subjected to experimental manipulations (Roach 1993), will deeper insights to be gained into these questions.

### 7. What Factors Have Driven Deep Evolutionary Differences in the Relationships Between Mortality Rates in Plants and Their Metabolism?

Marbà, Duarte & Agustí (2007) have shown that the allometric scaling between mortality rates and sizes differs for aquatic and land plants; the latter display a steeper reduction in mortality for an increasing unit of size than aquatic plants. Similar trends have also been noted across the tree of life (Sibly, Brown & Kodric-Brown 2012). Explanations for these differences in the relationships between the speed of life and size may depend upon architectural and chemical constraints that were under divergent selection in deep evolutionary time (Finch 1990). The same findings may apply to vascular *vs*. nonvascular plants, and perhaps to other evolutionary splits, such as that between monocots and dicots (Wright *et al*. 2004). We predict that better understanding of age-specific trajectories will shed important light on these relationships.

### 8. Can Plants Avoid Whole-Individual Senescence Through Fine-Tuning Their Allocation of Resources Between Survival and Reproduction?

In other words, if a plant species shows senescence for age-specific survivorship (*l*_*x*_), does it necessarily also show senescence for age-specific reproduction (*m*_*x*_)? The outcome of the *l*_*x*_ and *m*_*x*_ product affects the reproductive value of the individual and ultimately its force of selection (Hamilton 1966). Yet, some studies have noted declines in one term and not in the other, for example, in some commercially important tree species (Harper 1977). The aster *Tanacetum vulgare* (Münzbergová *et al*. 2005) shows declines in ramet survival but increases in fecundity with age, whereas the desert borage *Cryptantha flava* exhibits declines in fecundity but increases in survival with age (R. Salguero-Gómez & B. B. Casper, unpubl. data). The possibility of decoupling these two fitness components, which does not seem to occur in animals (Jones *et al*. 2008), requires closer examination.

### 9. Can Dispersal Patterns and Facilitative Interactions Extend Life Span in Plants as in Some Social Animals?

Humans are able to exhibit an extended postreproductive phase of life. It has been argued that this life-history strategy has evolved due to the fitness benefits of the care by grandmothers on their grandchildren (Lahdenperä *et al*. 2004). Local clustering of kin may yield an extended postreproductive life span in cetaceans and in primates as well (Johnstone & Cant 2010). Plants may not be ‘social’ in the animal sense, but they may cluster with their kin due to localized seed dispersal and clonal propagation (Kalisz *et al*. 2001; Hardy *et al*. 2006). In plants, the persistence of older individuals may keep mutualistic microbes at higher concentrations than might be possible with high recruitment, as old plants may provide more stable habitat than seedlings via, for instance, mycorrhizal networks (Bever *et al*. 2009). Would an extended life span yield greater reproductive success in younger, related plant individuals, particularly in increased kin contact with mutualistic microbes?

### 10. Are We Using Appropriate Demographic Metrics to Study Whole-Plant Senescence?

Demography is the science of the living and the reproductive. In measuring a state variable to inform plant population models, one aims to choose the variable that most closely correlates with survival and reproduction. Tree demographic models are typically based on diameter at breast height (d.b.h), but d.b.h measures tissue that is mostly dead. Would predictions of tree senescence based on models be different if we used a more dynamic, living tree part, such as the canopy? Lamar & McGraw's (2005) on the demography of *Tsuga canadensis* suggests that it would. The authors built matrix models based on d.b.h and on GIS canopy photography; the output of both models for the same population differed significantly. While the trunk of a tree is physiologically constrained to remain the same size or to grow slowly between years (but see Holder 2008), declines in survival and reproduction have been reported for old trees that have also lost part of their canopy (Sprugel 1976). On a broader scale, we argue that size has become the usual state variable of choice in plants and other modular organisms (e.g. corals) at the expense of the exploration of age effects in such species. Naturally, this choice has been made out of convenience (measuring size is easier than measuring age) or necessity, as anatomical markers of age are not available for most species of herbs and some trees, and when they are available, their destructive sampling is incompatible with demographic censuses (but see García, Dahlgren & Ehrlén 2011).

In summary, the field of research into senescence is replete with questions for which ecologists who study plant population dynamics are ideally positioned to provide the answers. Moreover, a better understanding of senescence will improve our understanding of plant ecology and evolution. We argue that early (Goodman 1969) and recent modelling developments (Caswell & Salguero-Gómez 2013; Metcalf *et al*. 2013) may prove particularly useful when age can be estimated and that size and age may interact in influencing population dynamics (Shefferson & Roach 2013). In addition, a vast amount of demographic information already exists in the form of projection matrix models (Salguero-Gómez & de Kroon 2010; Salguero-Gómez 2013). These data are an immensely valuable but as yet greatly underexplored resource for comparative analyses to address important questions about senescence across the plant kingdom. At the same time, we need more ‘greenhouse/field rats’ on which to explore questions about whole-plant senescence. The *Plantago* (Shefferson & Roach 2013), *Borderea* (Morales *et al*. 2013), *Silene* and *Astragalus* (Tuomi *et al*. 2013) species used in research reported in this Special Feature are excellent herbaceous model systems – but they are not the only suitable ones (e.g. *Ophrys sphegodes*; Hutchings 2010). Another obvious choice is *Arabidopsis*, in which the whole genome has been sequenced (Marra *et al*. 1999) and the genes responsible for whole-plant senescence identified (Guo & Gan 2011). In addition, research into plant senescence is in need of long-term data in general and of data on ‘nonconventional’ demographic study species such as nonvascular plants (mosses, liverworts, hornworts and algae), lower plants (ferns) and woody species.

## Conclusions

The field of senescence is by historical inertia dominated by research on humans. The main emphasis of research into senescence to date has been on whether and how humans can slow it down, and even postpone it (Bartke *et al*. 2001; Crews 2003; Maier *et al*. 2010; Vaupel 2010). We argue that there are at least three reasons why human demographers, animal ecologists and plant population ecologists should work together. First, all three parties are currently asking the same questions, although perhaps with different terminology. Human demographers are interested in how cultural background and migration affect population dynamics and senescence rates (Crews 2003), whereas animal and plant population ecologists are interested in maternal effects and dispersal (Sutherland *et al*. 2013). Second, senescence is a phenomenon caused by evolutionary processes, and the comparative method has previously proved useful in ascertaining the ecological and physiological processes necessary for its evolution (Nunn 2011). Research that ignores taxonomic boundaries will advance our understanding of evolutionary senescence. Thirdly, for decades, animal demographers have been developing robust statistical tools to explore the evolution of senescence that account for differences between individuals within populations (Vaupel, Manton & Stallard 1979; Vaupel 1990) with imperfect long-term data (Colchero, Jones & Rebke 2012). All of these techniques could prove useful in the plant world too, particularly in the examination of long-lived species. Furthermore, we argue that the transfer of knowledge between these research factions should be tri-directional. For instance, the work by Caswell & Salguero-Gómez (2013) in this Special Feature introduces a novel method for quantifying selection gradients on age and stage in plants that is equally applicable to the analyses of data from humans and the rest of the animal kingdom.

Whole-plant senescence is basically a demographic phenomenon. Because demography channels adaptive evolution (Metcalf & Pavard 2007), senescence must necessarily affect most if not all aspects of ecology and evolution. Holt (1996) introduced an outstanding example of the way in which consideration of senescence may improve our understanding of key ecological concepts such as source-sink dynamics. In our opinion, a mechanistic understanding of the evolution or lack of senescence in plants will only be achieved when evolutionary theories are supplemented with data from ecological field experiments. Plant ecologists are in a privileged position to explore the conditions for the evolution of senescence and to make plants count, particularly given the strong possibility that some plants may not be able to do so for themselves.
